# Genetic rescue stabilizes diversity in small isolated populations of Bonneville cutthroat trout

**DOI:** 10.1038/s41598-026-65885-8

**Published:** 2026-08-31

**Authors:** Tanner S. Van Orden, Peter C. Searle, Andrea L. Kokkonen, Dennis K. Shiozawa, Jonathan S. Reynolds, R. Paul Evans

**Affiliations:** 1 Department of Fisheries, University of Alaska, Fairbanks, AK USA; 2 Department of Ecology and Evolutionary Biology, Cornell University, Ithaca, NY USA; 3 Department of Microbiology and Molecular Biology, Brigham Young University, Provo, UT 84602 USA; 4 Department of Biology, Brigham Young University, Provo, UT USA; 5 National Park Service, Great Basin National Park, Baker, NV USA

**Keywords:** Low-coverage whole-genome sequencing, Cutthroat trout, Population genetics, Nucleotide diversity, Ecology, Ecology, Evolution, Genetics, Zoology

## Abstract

Genetic diversity loss due to anthropogenic factors is occurring rapidly on a global scale, putting many species at risk of extirpation and extinction. Different management strategies have been developed to slow this loss; however, it is often unknown whether these strategies reach their intended goals. In this study, we evaluate population structure and changes in nucleotide diversity (π) in isolated populations of Bonneville cutthroat trout (*Oncorhynchus clarkii utah)* from the Snake Range (Nevada, USA). Starting in the 1990s, three of these populations were used to reestablish populations in the Snake Range because many of the historic populations were extirpated. Some populations were stocked using a single-source and others were stocked using multiple-sources. Using low-coverage whole-genome sequencing coupled with historic samples (2003–2010) and contemporary samples (2019–2022), we find that single-source populations lost nucleotide diversity while mixed-source populations maintained nucleotide diversity. Further, source populations used to restore populations throughout the Snake Range lost the most nucleotide diversity over the time span evaluated. Our findings provide insight into how small, isolated populations can be managed to maintain genetic diversity.

**Supplementary Information:**

The online version contains supplementary material available at https://doi.org/10.1038/s41598-026-65885-8.

## Introduction

Genetic diversity loss is occurring rapidly on a global scale, putting many species at risk of extinction^1^. Anthropogenic factors, including land-use, overharvesting, invasive species, and ecological disruption, are driving this loss by reducing gene flow, fragmenting habitat, and altering community dynamics^2–6^. Low genetic diversity can ultimately lead to reduced adaptive potential, loss of life-history variation, and inbreeding depression, all of which impact the viability of populations^7^. Thus, monitoring genetic diversity at multiple scales is critical for predicting the future survival of populations, species, and ecosystems^8^.

Management strategies, including species reintroduction, legal protection, habitat restoration, breeding, supplementation, and invasive species removal, have been developed to combat the loss of genetic diversity^1,9,10^. However, it is often unknown whether these strategies reach their intended goals^10,11^. A recent meta-analysis by Shaw et al.^1^ evaluated genetic diversity loss in 628 species and found that some strategies (e.g., ecological restoration, invasive species removal, population control, and supplementation) maintained or increased genetic diversity, while other strategies (e.g., legal protection, breeding, and temporary resources) were associated with loss of genetic diversity. Furthermore, another study found that in 20% of instances when intervention took place, biodiversity declined more than expected^9^. Ultimately, these studies underscore the importance of carefully evaluating whether a proposed management strategy will effectively maintain genetic diversity.

Understanding the loss of genetic diversity in freshwater systems is particularly important because they support one-fourth of global vertebrate biodiversity and ~ 6% of all described species, despite covering less than 1% of Earth’s surface^4,12^. Freshwater systems are particularly at risk for genetic diversity loss because they are often small, relatively disconnected, and regularly subject to anthropogenic impacts such as the introduction of non-native species, altered flow regimes, and habitat pollution^4,13,14^. One approach to preserve at-risk lineages is to create population replicates using either a single source or multiple sources. Single-source population replicates preserve population-level diversity and create redundancy which can protect the complete loss of distinct evolutionary lineages. However, single-source population replicates can also suffer from low genetic diversity which can ultimately impact the resilience of these populations. Another approach is to create populations from multiple sources (henceforth mixed-source population replicates) to protect species-wide genetic diversity. The disadvantage of this approach is that such populations no longer represent the same unique genetic lineages that they were sourced from.

**Fig. 1:**
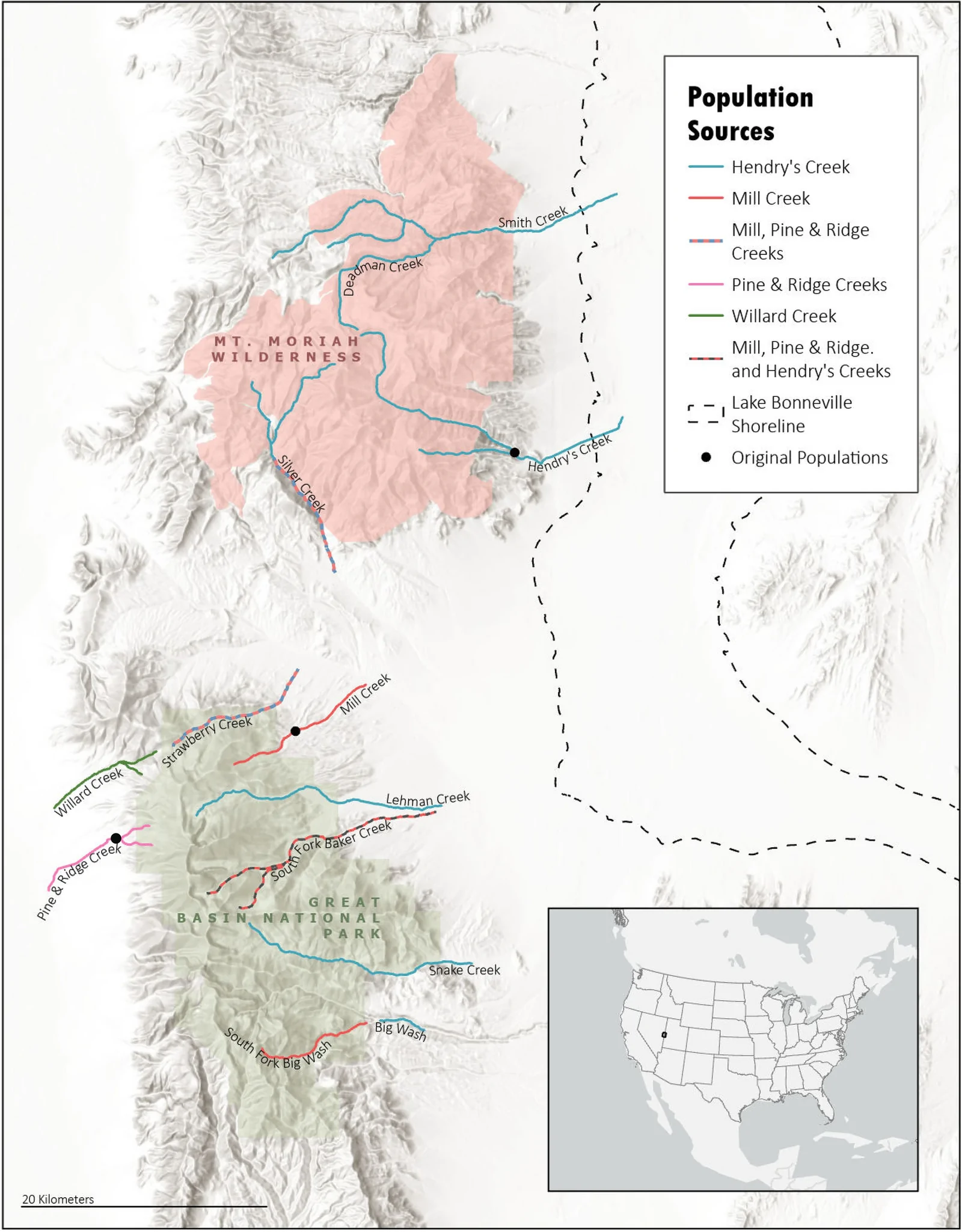
Study area including creeks colored by stocking source. Pure source populations used to restore other creeks are marked with a circle. The maximum extent of Lake Bonneville is shown as a black dotted line.

Bonneville cutthroat trout (*Oncorhynchus clarkii utah*, BCT) is a subspecies of cutthroat trout found within the Bonneville Basin in the intermountain West of the USA^15^. BCT populations from the Snake Range in Nevada, USA, were naturally isolated from other BCT populations following the desiccation of Lake Bonneville ~ 11,200 years ago^16–19^, (Fig. 1). Because of their location on the western periphery of Lake Bonneville’s maximum extent^20^ and thus their long-term isolation, these populations are genetically distinct from other BCT populations throughout the Bonneville Basin^21–23^. Furthermore, like many salmonids, the range of this subspecies has been further reduced due to anthropogenic activities, resulting in highly fragmented and isolated populations throughout the subspecies’ range^18,24,25^.

Bonneville cutthroat trout were historically found in 12 endorheic streams in the Snake Range. However, due to 1) competition with non-native salmonids, primarily brook trout (*Salvelinus fontinalis*) and brown trout (*Salmo trutta*), 2) hybridization with rainbow trout (*Oncorhynchus mykiss*) and/or other non-native cutthroat trout (*Oncorhynchus clarkii spp.*), and 3) heavy cattle grazing, BCT were extirpated from 10 of these 12 historic streams leaving only Mill Creek and Hendry’s Creek with pure populations. Additionally, pure populations were identified in Pine & Ridge Creeks (Pine & Ridge Creeks are a single connected watershed and thus treated as a single stream in this paper), and Willard Creek, all of which are outside the native range of this subspecies. These populations may have been transferred into these systems through the Osceola Ditch, which carried water from the Bonneville Basin to the Snake Valley for mining purposes^26,27^, (Personal Communication, Jonathan Reynolds). Because BCT populations were extirpated from many streams, fish were reintroduced into 10 streams within the Snake Range starting in 1997 (Supplementary Table 1). Streams were either stocked with fish from a single source or from multiple source populations using Hendry’s, Mill, and Pine & Ridge Creeks^28^, (Personal Communication, Jonathan Reynolds; Table 1; Fig. 1).

**Table 1:** All populations evaluated in this study, including the year sampled, geographic location in the Snake Range, status, source strain(s), sample size and nucleotide diversity (π). Source represents a pure population used for stocking other streams.

Population	Year	Origin	Location	Status	Source strain(s)	Sample size (n)	Nucleotide diversity (π)
Hendry’s Creek	2009	Historic	North	Source	Native	7	0.238
Hendry’s Creek	2019/2021	Contemporary	North	Source	Native	42	0.215
Mill Creek	2009	Historic	South	Source	Native	8	0.283
Mill Creek	2022	Contemporary	South	Source	Native	18	0.264
Pine & Ridge Creeks	2022	Contemporary	South	Source	Undocumented	29	0.168
Willard Creek	2003	Historic	South	Unknown	Undocumented	5	0.184
Willard Creek	2022	Contemporary	South	Unknown	Undocumented	30	0.200
Silver Creek	2010	Historic	North	Mixed Source	Hendry’s, Mill, and Pine & Ridge Creeks	10	0.20
Silver Creek	2022	Contemporary	North	Mixed Source	Hendry’s, Mill, and Pine & Ridge Creeks	37	0.215
South Fork Baker Creek	2009	Historic	South	Mixed Source	Hendry’s, Mill, and Pine & Ridge Creeks	9	0.260
South Fork Baker Creek	2021	Contemporary	South	Mixed Source	Hendry’s, Mill, and Pine & Ridge Creeks	26	0.264
Big Wash Creek	2010	Historic	South	Mixed Source	Hendry’s and Mill Creek	9	0.254
Big Wash Creek	2022	Contemporary	South	Mixed Source	Hendry’s and Mill Creek	27	0.255
Strawberry Creek	2009	Historic	South	Mixed Source	Mill Creek and Pine & Ridge Creeks	10	0.291
Strawberry Creek	2022	Contemporary	South	Mixed Source	Mill Creek and Pine & Ridge Creeks	29	0.291
Deadman Creek	2009	Historic	North	Single Source	Hendry’s Creek	9	0.211
Deadman Creek	2022	Contemporary	North	Single Source	Hendry’s Creek	31	0.198
Lehman Creek	2020	Contemporary	South	Single Source	Hendry’s Creek	27	0.218
Smith Creek	2009	Historic	North	Single Source	Hendry’s Creek	8	0.220
Smith Creek	2022	Contemporary	North	Single Source	Hendry’s Creek	34	0.213
South Fork Big Wash Creek	2010	Historic	South	Single Source	Mill Creek	10	0.266
South Fork Big Wash Creek	2022	Contemporary	South	Single Source	Mill Creek	48	0.248
Snake Creek	2022	Contemporary	South	Single Source	Hendry’s Creek	52	0.211

In this paper, we use variable sites extracted from low-coverage whole-genome sequencing of historic and contemporary samples to investigate population genetic structure and changes in nucleotide diversity (π) across a ~ 15-year period_._ Our analyses revealed that different management strategies had different outcomes on nucleotide diversity, highlighting the importance of carefully monitoring at-risk populations.

## Results

### Variable site identification

All 504 samples used in this study had ~ 2 GB of sequencing data. Average read depth after mapping to a Greenback cutthroat trout genome was 0.52 × coverage (Supplementary Table 2). ANGSD identified 6,788,441 variable sites that passed filtering. After removing sites in linkage disequilibrium, 824,255 variable sites remained (Supplementary Fig. 1).

### Principal components analysis

**Fig. 2:**
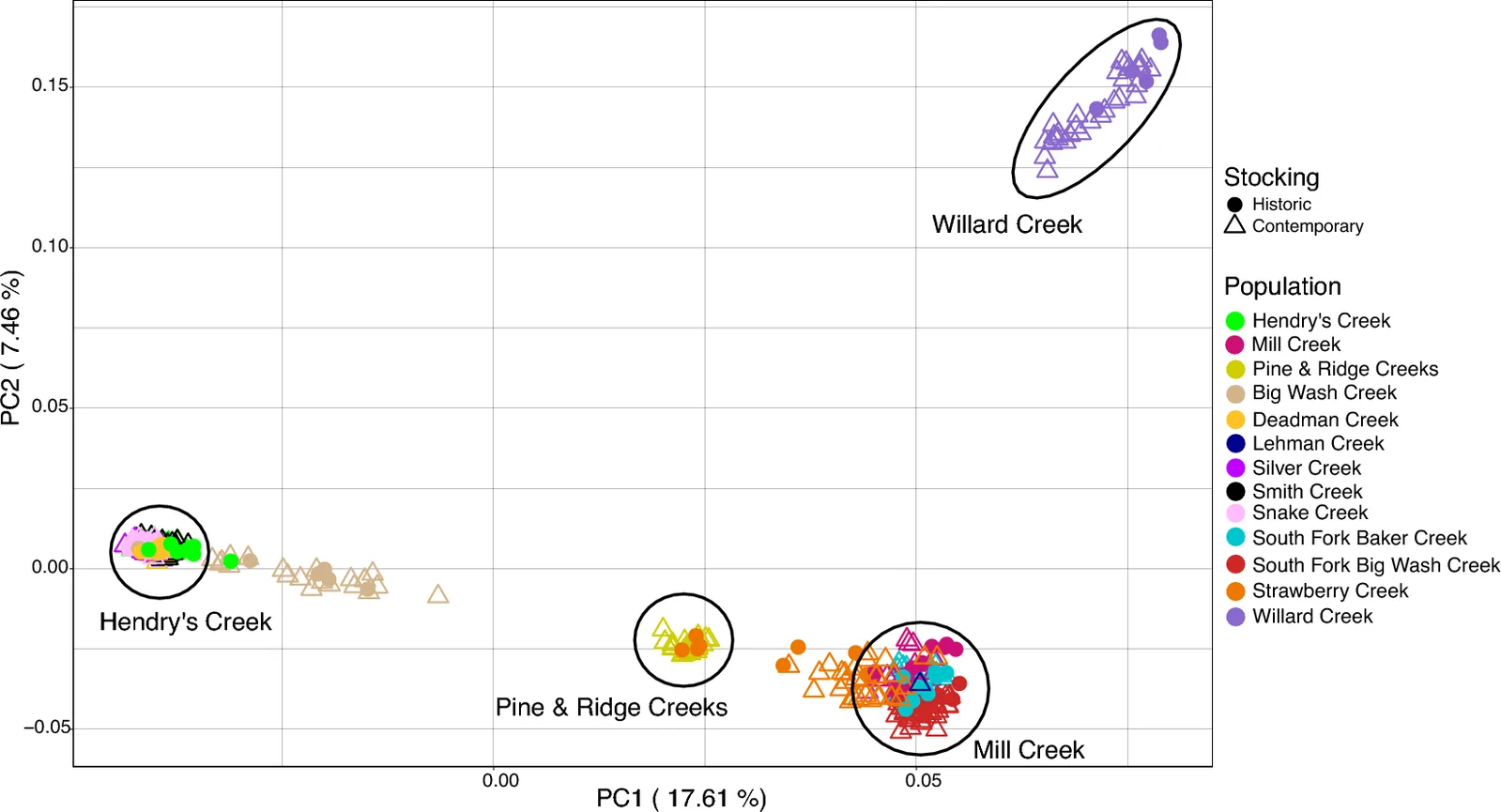
Principal component analysis of all samples used in this study. The four original pure populations (Hendry’s, Mill, Pine & Ridge, and Willard Creek(s)) are circled. Historic samples (2003–2010) are shown as filled circles, while contemporary samples are shown as open triangles (2019–2022).

All source populations, including Mill Creek, Hendry’s Creek, and Pine & Ridge Creeks, formed distinct clusters (Fig. 2). Additionally, individuals from Willard Creek clustered separately from all populations evaluated. Single-source populations grouped with their source populations, while mixed-source populations either grouped amongst and/or between their source populations (Fig. 2). For example, one sample from Lehman Creek clustered with Mill Creek, while the remaining samples clustered with Hendry’s Creek.

### *F*_*st*_

**Fig. 3:**
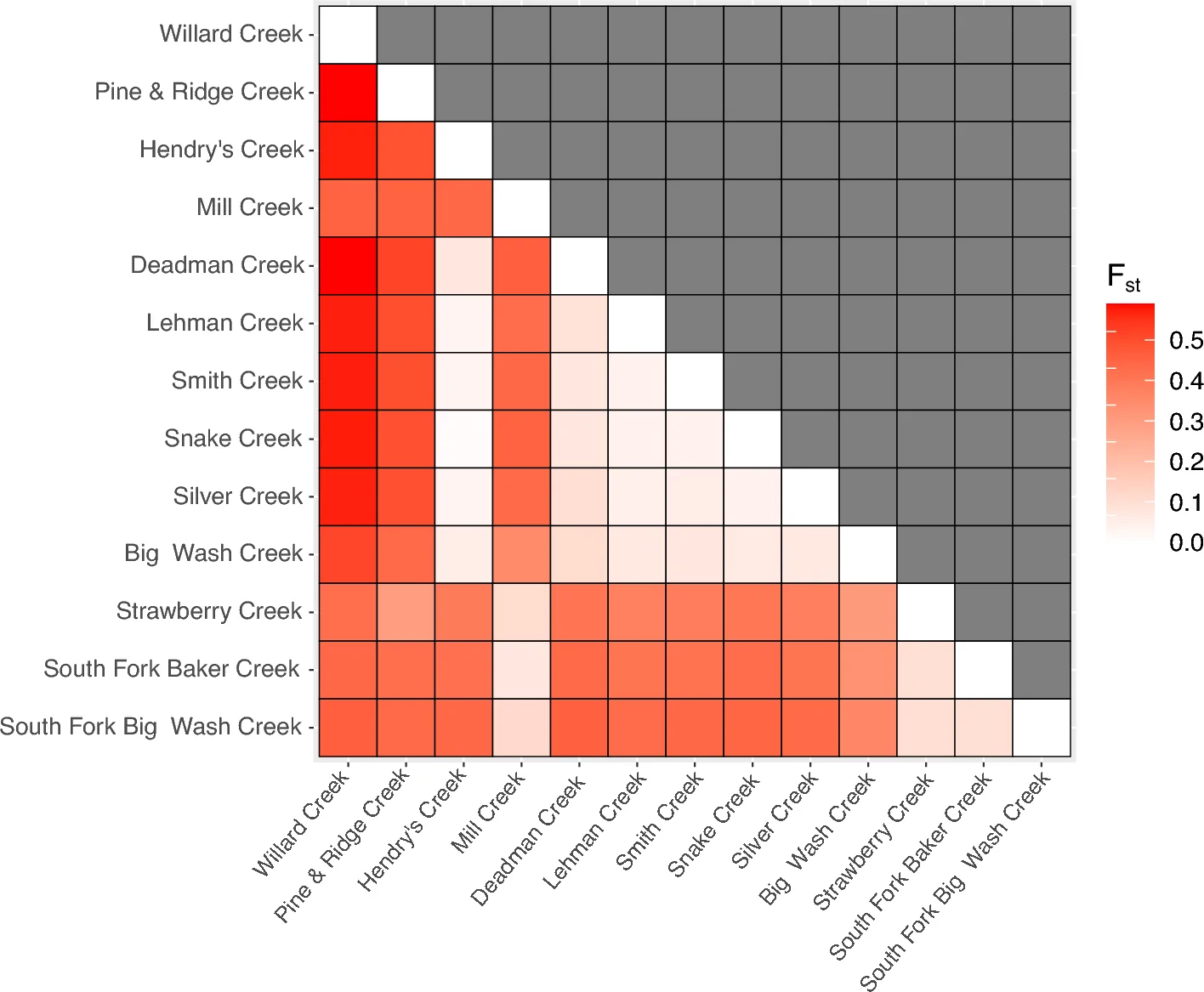
F_st_ population differentiation.

F_st_ values ranged from 0.011 to 0.590. Willard Creek was the most differentiated population of all populations evaluated (Fig. 3; Supplementary Table 3). Single-source populations had lower F_st_ values when compared to their source populations. Mixed-source populations were more similar to one of their source populations than to the other(s). For example, Big Wash Creek, which was stocked with fish from Hendry’s Creek and Mill Creek, was more similar to Hendry’s Creek (0.055) than Mill Creek (0.348; Fig. 3).

### Nucleotide diversity (π)

**Fig. 4:**
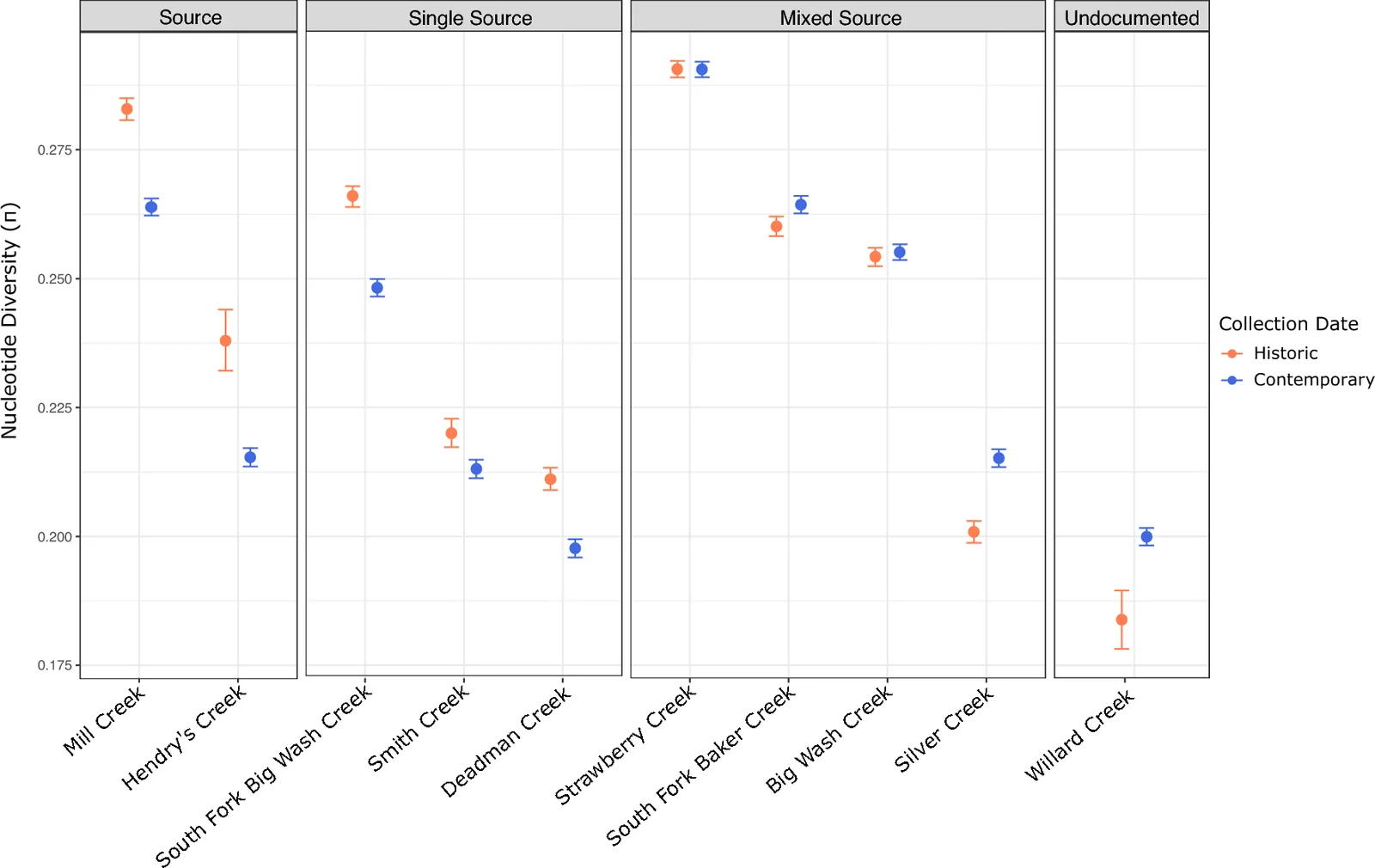
Nucleotide diversity (π) estimates for historic and contemporary samples. Error bars represent 95% confidence intervals calculated with 10,000 bootstraps using 10,000 bp statistically independent windows.

Average nucleotide diversity (π) in the historic samples ranged from 0.184 in Willard Creek to 0.291 in Strawberry Creek, and in the contemporary samples ranged from 0.168 in Pine & Ridge Creeks to 0.291 in Strawberry Creek (Table 1). Of the populations we had both historic and contemporary samples, source populations (Hendry’s and Mill Creeks) and single source populations (Deadman, Smith, and South Fork Big Wash Creek) lost nucleotide diversity, while mixed-source populations maintained (Strawberry and Big Wash Creeks) or gained genetic diversity (Silver and South Fork Baker Creeks) during the study period (Fig. 4). Our subsampled dataset showed similar results (source and single-source populations lost genetic diversity, while mixed-source populations maintained or gained genetic diversity) with one exception; Smith Creek showed a minimal increase in contemporary nucleotide diversity (0.2212) compared to historic nucleotide diversity (0.220; Supplementary Table 4).

## Discussion

Utilizing low-coverage whole-genome sequencing, we evaluated population genetic structure and nucleotide diversity (π) in populations of Bonneville cutthroat trout (BCT) in the Snake Range (Nevada, USA) across 5 to 10 generations (~ 15 years). Our results suggest that stocking strategy influenced nucleotide diversity. Specifically, streams stocked with a single source lost nucleotide diversity, whereas those stocked with multiple sources maintained or gained nucleotide diversity (Fig. 4). In addition, we found that source populations used for reintroductions in the Snake Range also lost nucleotide diversity. Although the number of populations per group (source, single-source, and mixed-source) in the historic samples limited statistical power, the consistent pattern of temporal changes in nucleotide diversity within each group suggests distinct trajectories. Altogether, these results highlight that management strategies with the same objective (e.g. preserving genetic diversity) often produce different conservation outcomes. Overall, our study suggests the importance of three key management strategies: 1) the use of single-source population replicates to preserve genetic diversity at the population level, 2) mixed-source population replicates to preserve diversity at the species level, and 3) the frequent monitoring of genetic diversity in broodstock and restored populations.

### Single-source population replicates preserve population level diversity

Isolated populations often represent unique genetic lineages and thus are high-priority populations for conservation management^29^. Unfortunately, isolated populations in freshwater systems are often at risk of extirpation due to increased intensity of droughts and fires, as well as the introduction of invasive species^30–32^. One management strategy to preserve population-level diversity and prevent the complete loss of distinct evolutionary lineages in these systems is to generate single-source population replicates of at-risk species^33,34^. The 2012 Whitewater-Baldy Fire in the Gila Wilderness (New Mexico, USA) is an example of successful implementation of this strategy. The fire impacted half of all Gila trout (*Oncorhynchus gilae*) populations and, unfortunately, three of the six affected populations were extirpated. In response to this loss, the Gila Trout management strategy now includes the replication of each relict population in geographically separate areas, reducing the probability that a single extreme event could wipe out an entire lineage^35^. Similar examples where refuge populations have been created to protect evolutionarily significant lineages include cutthroat trout^36,37^, redband trout^38^, western mosquitofish^39^, and pupfish^34,40^.

Using single-source population replicates has successfully preserved relict lineages of BCT in the Snake Range. Following the desiccation of Lake Bonneville ~ 11,200 years ago, Snake Range BCT became isolated from other BCT populations^16^. Without gene flow to counteract random changes in allele frequencies, genetic drift and local adaptation would have contributed to the distinct morphology observed in previous studies^22^, as well as the moderate-to-high F_st_ values and high levels of population genetic structure observed within our study (Fig. 2 and 3)^41^. This distinctiveness is one reason why single-source population replicates were created to preserve the three source streams in the Snake Range (Hendry’s Creek, five replicates; Mill Creek, one replicate; Pine & Ridge Creeks, one replicate).

Currently, these populations are at risk of extirpation due to an intensifying 1,200-year drought. This drought has led to repeated stream desiccations in source populations (Hendry’s and Mill Creek), reducing population size. It has also increased the frequency and intensity of fires, resulting in the complete loss of BCT in Hampton Creek in 2014 (only replicate of Pine & Ridge Creeks) and approximately 85% of BCT in Strawberry Creek in 2016 (Personal Communication, Jonathon Reynolds)^42,43^. While previous studies have found that populations can quickly evolve and diverge from their original source populations^34,40^, our population genetic structure analyses indicate that these populations retained distinct genetic patterns (Fig. 2 and 3) consistent with their respective source over the time period evaluated in this study. Thus, single-source population replicates are a practical approach to ensure that the evolutionary distinctiveness and heritage of a population is maintained and safeguarded.

### Mixed-source population replicates preserve species level diversity

Mixed-source population replicates serve a different but equally important role when compared to single-source population replicates: protecting as much species-wide genetic diversity as possible. While single-source population replicates ensure that the unique evolutionary history of a population is maintained through time, it does not always lead to populations with high genetic diversity^39,44^. Consistent with experimental, observational, and theoretical studies evaluating genetic rescue in salmonids as well as in other species^45–48^, our results show that mixing two or more sources of BCT led to comparatively higher and more stable nucleotide diversity in mixed-source populations relative to single-source populations (Fig. 4). This is especially promising because these populations maintained nucleotide diversity in face of drought, and, in the case of Strawberry Creek, a fire in 2016 that almost extirpated the population (Personal Communication, Jonathan Reynolds). This approach continues to be utilized widely both in cutthroat trout and other salmonids in hopes of improving resilience in isolated populations^46,49–51^.

Although we were able to evaluate how genetic diversity changed in mixed-source populations over time, we were unable to determine whether these changes improved fitness and resilience. Increased genetic diversity through genetic rescue can maintain adaptive potential, reduce inbreeding depression, and promote long-term resilience of populations with low genetic diversity^46,50,51^. However, it can also have negative impacts by eroding unique alleles in locally adapted populations, as well as introducing deleterious alleles, ultimately resulting in outbreeding depression^52^. For salmonids, examples exist of both outcomes^46,50,51,53–55^, illustrating that it is important to evaluate both metrics to determine whether specific conservation outcomes are being achieved.

### Maintaining the genetic integrity of source populations

We evaluated temporal changes in nucleotide diversity within source populations, focusing on Hendry’s Creek and Mill Creek because historic samples were unavailable for Pine & Ridge Creeks. A substantial number of individuals were removed from these small populations beginning in the 1990 s to restore BCT populations throughout the Snake Range. Prior to historic sampling, 600 fish were taken from Hendry’s Creek and 178 from Mill Creek, followed by additional removals during contemporary sampling periods (224 fish from Hendry’s Creek and 65 fish from Mill Creek). Given their limited census sizes and the large number of individuals removed, we anticipated that source populations would exhibit the lowest nucleotide diversity in our dataset. Instead, both showed intermediate to high diversity relative to other populations (Table 1; Fig. 4). However, temporal comparisons revealed pronounced declines: nucleotide diversity decreased by 9.66% in Hendry’s Creek and 7.20% in Mill Creek over roughly a decade, representing some of the largest losses observed among all evaluated populations. These declines likely reflect the combined effects of demographic contraction and fish removal; however, their relative contributions cannot be disentangled with our available data.

Two events could have contributed to these results: (1) intense drought causing repeated desiccation of the lower reaches of the streams, and (2) removal of 224 fish from Hendry’s Creek (2019–2020) and 65 fish from Mill Creek (2010–2012). The current drought in the Great Basin intensified between 2020 and 2021, resulting in below-average stream flows in the Snake Range^56^. For example, in 2021, the lower 0.97 km of Mill Creek were desiccated, which left only 0.64 km of remaining BCT habitat, leading to a large reduction in population size (Personal Communication, Jonathan Reynolds). Ultimately, reduced flows in headwater streams can decrease cutthroat trout populations by as much as 50%, which can dramatically reduce genetic diversity. The loss of nucleotide diversity in both source and single-source populations could be attributed to drought. Similarly, studies have shown that removal of individuals from a population can lead to nucleotide diversity loss^57^. Thus, the comparatively higher loss in source populations could suggest fish removal impacted nucleotide diversity in these populations in addition to drought. While we are unable to determine whether these events had a direct impact on these populations, the magnitude of diversity loss underscores the need for routine genetic monitoring of source populations to ensure that restoration efforts do not inadvertently erode the genetic integrity of the very lineages they aim to preserve.

Other useful metrics such as heterozygosity and effective population size (Ne) would provide additional insight into these populations. However, uneven sample size between historic and contemporary samples, as well as the use of extremely low-coverage whole-genome sequencing (~ 0.52 × coverage), limited the analyses we could complete^58^. These limitations also did not allow for the calculation of absolute nucleotide diversity, and as such our nucleotide diversity results are only appropriate for the comparison of nucleotide diversity between the populations evaluated in our dataset^58,59^. Additionally, we observed variation within our contemporary nucleotide diversity estimates after subsampling (Supplementary Table 4). However, the general trends of source and single-source populations losing genetic diversity, and mixed-source populations maintaining or gaining genetic diversity were still apparent. This variation in our estimates likely reflects family structure/sibling relationships, which is expected in small, isolated populations and is known to impact estimates of genetic diversity^60^.

## Conclusion

As anthropogenic impacts continue to threaten isolated populations of fishes and other taxa, evaluating whether management strategies achieve conservation outcomes is critical for preserving these species. The three strategies discussed above, maintaining single-source population replicates, creating mixed-source population replicates, and sustaining genetic integrity of original source populations, all represent important ways to protect genetic diversity. Creating additional single-source replicates of Mill Creek and Pine & Ridge Creeks will preserve the legacy of these unique lineages, while introducing additional mixed-source population replicates will preserve species-level diversity in BCT in the Snake Range. Ultimately, conserving genetic diversity within and among populations is fundamental to maintaining evolutionary potential and ensuring the long-term persistence of species.

## Methods

### Sampling

Fin clips were collected from Bonneville cutthroat trout (BCT) in the Snake Range by personnel from Great Basin National Park and the Nevada Department of Wildlife using electrofishing in 2003, 2009, 2010, and 2019–2022. 11 of the 12 populations within the native range of BCT in the Snake Range were sampled at multiple locations to ensure we utilized a broad set of samples. We did not analyze Hampton Creek because a high-intensity fire in 2014 eliminated its BCT population (Personal Communication Jonathon Reynolds). The samples that were used in this study represented both historic (2003, 2009, and 2010; n = 84; 10 streams) and contemporary (2019- 2022; n = 430; 11 streams) samples (Table 1). We chose to include Silver Creek as an additional mixed-source population in our analyses because Silver Creek was stocked with fish primarily from Snake Creek (source population Hendry’s Creek) as well as a small number of fish from Strawberry Creek (source populations Mill Creek and Pine & Ridge Creeks). Although we did not have historic samples for this population, we did have historic samples from Snake Creek, allowing us to make this additional comparison (Fig. 1; Supplementary Table 1). The difference between the number of historic and contemporary samples is due to increased sampling for genetic studies in recent years, plus limited archived historic samples. All fin clips were stored in ethanol.

### DNA isolation and sequencing

Whole genomic DNA was isolated using DNeasy® tissue kits (Qiagen, Germantown, MD, USA) following the manufacturer’s recommended protocol. DNA quantity and purity were verified using a NanoDrop One (Thermo Fisher Scientific, Waltham, MA, USA). Novogene America (Davis, CA, USA) prepared the libraries using the Illumina Nextera DNA Flex kit and sequenced them on an Illumina Hi-Seq 2500 (Illumina, San Diego, CA, USA) using paired-end reads (2 × 150 bp). DNA libraries were randomly distributed across 4 lanes and ~ 2 GB of data were sequenced for each sample.

### Quality control and read mapping

Sequence quality was assessed with FastQC v.11.8^61^. The raw reads were trimmed with Trimmomatic v.0.39 1^62^ and then mapped to a Greenback cutthroat trout genome (*Oncorhynchus clarkii spp.;* In Prep) using Bowtie2 v.2.5.2^63^. Overlapping reads were trimmed and clipped using Picard Mark Duplicates v3.1.1 and BamUtil clipOverlap v1.0.15^64,65^.

### Variable site identification

Genotype likelihoods were extracted from all samples using ANGSD v.940 with the SAMtools genotype likelihood model (-GL 1). We followed the best practices for downstream analyses pertaining to population genetic structure and genetic diversity as outlined in Hemstrom et al.^66^. Specifically, major and minor alleles were inferred (-doMajorMinor 1), and minor allele frequencies were estimated (-doMaf 1). Only sites that were present in at least five individuals (-minInd 5) that had a minor allele frequency greater than or equal to 0.05 (-minMaf 0.05) were included in our analyses. To reduce errors, we used a minimum base quality score of 30 (-minQ 30) and a minimum mapping quality score of 40 (-minMapQ 40). Reads marked as bad by SAMtools (-remove_bads 1), reads that mapped to more than one genomic region (-uniqueOnly 1), and read pairs not properly paired (-only_proper_pairs 1) were not included in our analyses^59^. Sites in linkage disequilibrium were filtered using an R^2^ value of 0.2 and a window size of 10,000 bp with ngsLD v1.2.0^67^ to ensure all sites were statistically independent.

### Population genomic analyses

Site allele frequency likelihoods were calculated (-doSaf 1) separately for each population using the same previously identified variable sites that were not in linkage disequilibrium. Historic and contemporary samples from the same population were treated as separate populations. Site frequency spectra were calculated for each population from the site allele frequency likelihoods using realSFS in ANGSD v.940. Nucleotide diversity (π) for each population was also calculated from the site frequency spectrum using ANGSD v.940 (saf2theta, do_stat) using a non-overlapping 10,000-nucleotide sliding window. Output files from saf2theta were read into R using the ANGSDR package, and the weighted mean nucleotide diversity was calculated using the survey package in R^68^. We generated weighted 95% confidence intervals for our nucleotide diversity estimates using 10,000 bootstrap replicates of each non-overlapping statistically independent 10,000 bp nucleotide window. Including confidence intervals allowed us to document uncertainty in our genetic diversity estimators due to different sample sizes and sequencing depth between contemporary and historic samples. Additionally, to evaluate how unequal sample sizes between historic and contemporary samples affected our nucleotide diversity estimates, we subsampled all contemporary populations to the same sample size as their corresponding historic population and re-estimated nucleotide diversity (Supplementary Table 4). Because these nucleotide diversity estimates were similar to our previous estimates, we chose to include all the contemporary samples for subsequent analyses.

2D site frequency spectrums were calculated using realSFS to compare population genetic structure, as well as calculate F_ST_ values. Additionally, we ran a principal components analysis (PCA) to compare BCT populations in the Snake Range. Principal components were calculated by taking the eigenvectors of the covariance matrices generated by ANGSD using R^69,70^.

## Supplementary Information


Supplementary Information 1.
Supplementary Information 2.
Supplementary Information 3.
Supplementary Information 4.


## Data Availability

The code underlying the genetic analyses in this article is available on GitHub at the link [[https://github.com/Tannervanorden/Great_Basin_NP_Cutthroat] (https://github.com/Tannervanorden/Great_Basin_NP_Cutthroat)]. The DNA dataset generated and analyzed during the current study are available on GenBank under BioProject accession PRJNA1431531 and BioSample accessions SAMN56313842 to SAMN56314393.
