# Supplementary material for: Genetic rescue stabilizes diversity in small isolated populations of Bonneville cutthroat trout
Source: Sci Rep. 2026 Aug 31;16:27233. doi: 10.1038/s41598-026-65885-8 (PMC13530152; doi:10.1038/s41598-026-65885-8)
Supplement: Supplementary file 1 [file 41598_2026_65885_MOESM1_ESM.docx]

Supplementary Figure 1. Density of sites mapped to the Greenback cutthroat trout genome used in this study.
